# Surgical site infection after delayed sternal closure in neonates with congenital heart disease: retrospective case-control study

**DOI:** 10.1186/s13052-021-01138-w

**Published:** 2021-09-08

**Authors:** Yuzhong Yang, Jie Wang, Lina Cai, Wei Peng, Xuming Mo

**Affiliations:** 1 Department of Cardiothoracic Surgery, Children’s Hospital of Nanjing Medical University, No.72 Guangzhou Road, Nanjing, Jiangsu Province, People’s Republic of China 210008; 2 Department of General Surgery and Ear-Nose-Throat, Children’s Hospital of Nanjing Medical University, Nanjing, China

**Keywords:** Delayed sternal closure, Surgical site infection, Congenital heart disease, Neonate

## Abstract

**Objectives:**

To determine the prevalence of surgical site infections (SSIs) in neonatal congenital heart disease patients undergoing delayed sternal closure (DSC) and evaluate risk factors for SSI.

**Methods:**

Hospital records of 483 consecutive neonates who underwent surgical intervention between January 2013 and December 2017 were reviewed, and perioperative variables were recorded.

**Results:**

We found that the prevalence of SSI was 87.5% when the body weight was less than 1500 g. When the operative age was between seven and 14 days, the probability of no SSI is about 93.9%. When the duration of the aortic cross-clamp was more than 60 min, the prevalence of SSI was 91.2%. The prevalence without SSI was 96.6% when the duration of DSC was less than 24 h. However, when the duration of DSC was more than 120 h, the prevalence of SSI was 88.9% (*p* = 0.000).

**Conclusions:**

With the prolongation of aortic clamping duration, the probability of occurrence of SSI increased in neonatal CHD with DSC. The age at operation and body weight are closely related to the occurrence of SSI in neonatal CHD patients with DSC.

## Introduction

The field of pediatric and neonatal cardiac surgery has witnessed major advances in the past 30 years [1]. At present, there is a strong emphasis on performing corrective operations early in life, often in the neonatal period itself, for forms of congenital heart disease (CHD) that can be corrected [2]. Most centers in China have reported excellent outcomes for neonatal cardiac surgery. However, postoperative health care–associated infections after pediatric cardiac surgery remain significant causes of morbidity and mortality [3–6]. Open-chest management with delayed sternal closure (DSC) is a surgical method used in the management of several conditions following cardiothoracic surgery [7]. This strategy avoids additional cardiac compression at a stage when myocardial swelling and increased lung water after cardiac surgery adds to the limited intrathoracic space in infants. However, DSC is not without potential infectious complications [8–11]. Few studies have explored the potential prevalence factors for SSIs in patients with DSC [12]. Delayed sternal closure (DSC) after cardiac surgery has been associated with infectious complications and a longer postoperative stay [13, 14]. So, we’re going to study it. The purpose of our study is as follows. a) Determining what factors are associated with this increased prevalence of infection may aid in prevention; b) Despite the high prevalence of bacterial infections in this patient population, there is limited data describing the epidemiology, prevalence factors, and patient outcomes and for those patients, this data may provide prognostic information, inform strategies for prevalence factors, and guide diagnostic workup and empiric antimicrobial therapy; c) The potential causative factors for SSI in this patient population have been as yet incompletely explored or characterized [15]. A clear understanding of outcomes and risk factors is essential because they may affect clinical decision making by cardiac surgeons and intensivists caring for these patients.

## Methods

### Case-control study

This work was conducted in line with the Strengthening the Reporting of Cohort Studies in Surgery (STROCSS) criteria [16]; it was a retrospective analysis of the hospital records of all neonates (age, ≤28 days) who underwent DSC after cardiac surgery between January 1, 2013, and December 31, 2017. This study was approved by the hospital’s Ethics Committee, and the need for informed consent was waived due to its retrospective nature. At our center, the decision to perform DSC is made by the attending surgeon in the operating room and is driven by intraoperative hemodynamic instability. At our center, we excluded patients who died within 48 h following surgery. Mortality was defined as in-hospital death or death within 30 days of cardiac surgery.

### Study design and primary outcome definition

A nested case-control study was conducted within the DSC cohort, comparing subjects with SSI to those without SSI. The SSI patients were diagnosed independently by two doctors, using published Centers for Disease Control and Prevention (CDC) criteria. SSI included incisional SSI and organ/space SSI. Control subjects were matched to case subjects in a 3:1 ratio based on year of surgery.

### Surgical technique

Delayed sternal closure was generally as follows [17–20]: 1) the sternum and subcutaneous tissue were kept open with a splint manually made from polypropylene syringe sutured to the sternum; 2) chest tubes and pacing wires were left in the mediastinal space; 3) the open chest was covered by Silastic patch sutured to the skin, sealed with synthetic adhesive, and additionally covered with a film dressing; 4) low negative pressure was maintained by continuous suction through chest tubes; and 5) chest washout with dressing change was performed in the CICU under aseptic measures as needed in cases of bleeding. Antibiotic prophylaxis was continued during the open chest management. The wound covered the sterile, thin, transparent film dressing. The dressing remained in place until sternal closure or other mediastinal procedures were required. The decision to close a sternum was at the discretion of the attending cardiac intensivist and surgeon. Typically, the sternum was closed when the patient was hemodynamically stable with adequate diuresis. DSC usually occurred in the operating room but was sometimes performed in the cardiac intensive care unit (CICU) under sterile conditions. A deep tissue sample was taken from the mediastinum for culture and sensitivity at the time of sternal closure in all cases. Chest tubes were cleared of all clots and repositioned. Following adequate hemostasis, the sternal edges were re-approximated with either interrupted stainless steel wires or braided absorbable sutures. The soft tissues and skin were closed using braided absorbable sutures in a running fashion. General anesthesia was provided during sternal closure. There were no changes to the standard techniques for DSC or sternal closure during the study period.

### Antibiotic prophylaxis and therapy

Patients received prophylactic antibiotic therapy with cefazolin (60–80 mg/kg a day) [20, 21], which was initiated during the induction of anesthesia and continued until 48 h following surgery. After discontinuation of this regimen, prophylactic antibiotic coverage for indwelling mediastinal and pleural chest tubes was initiated. Prophylaxis was continued until all chest tubes were removed. Antibiotic coverage was broadened based on clinical judgment for patients who developed fever or other clinical evidence of infection. We stopped using antibiotics until a state of stability of the disease was established and the clinical symptoms disappeared.

### Data collection

All data was retrospectively collected from the medical records of patients. This was a single-center, retrospective analysis. After all case and control subjects were identified, the primary author reviewed the medical record for all preoperative, intraoperative, and postoperative variables. Pertinent data collected included sex, weight, age at operation, genetic anomaly, prematurity (≤37 weeks [w]), time to find fetal congenital heart disease when prenatal detection was necessary, location of chest opening, type of surgical procedure, foreign material placed, duration of aortic cross-clamp, DSC, duration of DSC, duration of mediastinal chest tube, extracorporeal membrane oxygenation (ECMO), duration of mechanical ventilation, CICU length of stay, and hospital length of stay.

### Statistical analysis

Data is presented as frequencies, with percentage for categorical variables and median with interquartile range for continuous variables. Preoperative, intraoperative, postoperative, and clinical outcome variables were compared between cases and controls, using the χ^2^ test or Fisher’s exact test, as appropriate, for nominal variables. Variables found to be significantly associated with SSI in the univariate analyses (< 10) were investigated further by building multivariate logistic regression models to determine independent associations of risk factors with SSI. Results of the logistic regression are presented as odds ratio (OR) with 95% confidence intervals (CIs). A stepwise, multivariate model was constructed to evaluate risk factors. Variables that were not statistically significant (*p* > 0.05) were removed from the final multivariate model. All statistical analyses were performed with SPSS (version 23).

## Results

A total of 483 neonatal patients underwent DSC during the study period. Of these, 47 (9.7%) patients met CDC and National Healthcare Safety Network (NHSN) criteria for SSI (13 superficial incisional, four deep incisional, and 30 organ/space). There were 28 cases of mediastinitis and two cases of endocarditis within the group of patients with organ/space SSI. There was a significantly higher prevalence of SSI in patients with DSC. The prevalence of SSI among neonatal CHD patients who did not undergo DSC at our center during the study period was 1.1% (21 out of 1832), significantly lower than among the DSC cohort (*P* < 0.0001 from χ^2^ test). A total of 188 patients (47 cases and 141 controls) were included in the analysis. Demographic and related data is shown in Tables 1, ,2 and 3. There were no significant differences between case subjects and control subjects based on sex, genetic anomaly, and location of chest opening. In research on preoperative factors, we found that the prevalence of SSI was 87.5% when the body weight was less than 1500 g. However, when the body weight was more than 3000 g, it was 96.7% without SSI (Table 1). For age studies, we found that the probability of SSI was 84.6% when the operative age was less than 1 day and 84% when the operative age was less than 3 days. However, when the operative age was between 7 days and 14 days, the probability of no SSI was about 93.9%. In this group, patients more than 14 days old had no SSI (Table 1). The probability of mature neonates not developing SSI was about 93.2% compared with preterm infants (Table 1). In the study of intraoperative factors, we found that the SSI-free prevalence for patients who had no foreign material placed during cardiac surgery was about 94.7%, compared with patients who had material placed. The difference was statistically significant (*p* = 0.000, Table 2). We found no statistical difference in the prevalence of SSI when the duration of aortic cross-clamp was less than 45 min versus more than 45 min (*p* = 0.919, Table 2). But what is interesting is that the prevalence of SSI was 64.3% when the duration of aortic cross-clamp was more than 55 min (*p* = 0.000, Table 2). However, when the duration of aortic cross-clamp was more than 60 min, the prevalence of SSI was 91.2%. (*p* = 0.000, Table 2). In the study of postoperative factors, in comparison of one DSC with multiple DSCs, we found that patients with multiple DSCs were about 86% more likely to develop SSI (*p* = 0.000, Table 3). A majority (77.1%) of all patients requiring DSC had only a single period of DSC (Table 3). We found that the SSI-free prevalence was 96.6% when the duration of DSC was less than 24 h. However, when the duration of DSC was more than 120 h, the prevalence of SSI was 88.9% (*p* = 0.000, Table 3). In the analysis of whether to use ECMO, we found that the prevalence of SSI was about 88.6% when using ECMO, but absence of SSI was 89.5% when not using ECMO (*p* = 0.000, Table 3), and we found that the SSI-free prevalence was 93.9% when the duration of mechanical ventilation was less than 72 h. However, when the duration of mechanical ventilation was more than 120 h, the prevalence of SSI was 68.2% (*p* = 0.000, Table 3). Among patients with SSI, the mean length of hospital stay was 22.26 days (22.26 ± 4.341) and the mean length of intensive care unit stay was 11.64 days (11.64 ± 2.574); both were significantly greater than that of the control subjects (p = 0.000 and *p* = 0.000, respectively) (Table 4). Weight was highly correlated with prematurity (*p* = 0.000) and thus excluded from the multivariate model to avoid multicollinearity. Multivariate logistic regression analysis showed that weight and age at operation were significantly associated with increased prevalence of SSI (OR: 0.998 and 0.581, respectively; 95% CI: 0.997–0.999 and 0.474–0.711, respectively) (Table 5). The predominant organisms identified were coagulase-negative *Staphylococcus* species. The pathogens identified from positive mediastinal cultures included coagulase-negative staphylococci (36.17%), methicillin-susceptible *Staphylococcus aureus* (MSSA) (21.28%), *Enterococcus sp*. (12.76%), *Enterobacter cloacae* (8.51%), *Pseudomonas sp.* (6.38%), *Klebsiella oxytoca* (4.26%), *Serratia marcescens* (4.26%), and mycotic infection (6.38%).

**Table 1:** Comparison of preoperative characteristics of patients with and without surgical site infections

		All (*n* = 188)	Surgical site infection		χ^2^	*P* value
			No (*n* = 141)	Yes (*n* = 47)		
Sex	Male	92	71	21	0.454	0.5
	Female	96	70	26		
Weight	W ≤ 1500 g	8	1 (12.5%)	7 (87.5%)	43.262	0.000
	1500 g < w ≤ 2000 g	50	30 (60%)	20 (40%)		
	2000 g < w ≤ 2500 g	50	41 (82%)	9 (18%)		
	2500 g < w ≤ 3000 g	20	11 (55%)	9 (45%)		
	W > 3000 g	60	58 (96.7%)	2 (3.3%)		
Age at operation	≤1 d	13	2 (15.4%)	11 (84.6%)	111.572	0.000
	1<age ≤ 3 d	25	4 (16%)	21 (84%)		
	3<age ≤ 7 d	26	14 (53.8%)	12 (46.2%)		
	7<age ≤ 14 d	49	46 (93.9%)	3 (6.1%)		
	>14 d	75	75 (100%)	0		
Genetic anomaly	no	171	129 (75.4%)	42 (24.6%)	0.022	0.883
	yes	17	12 (70.6%)	5 (29.4%)		
Prematurity (≤37w)	no	133	124 (93.2%)	9 (6.8%)	80.606	0.000
	yes	55	17 (30.9%)	38 (69.1%)		

*P*<0.05, Chi squared test

**Table 2:** Comparison of intraoperative characteristics of patients with and without surgical site infections

		All (*n* = 188)	Surgical site infection		χ^2^	*P* value
			No (*n* = 141)	Yes (*n* = 47)		
Location of chest opening	CICU	34	27 (79.4%)	7 (20.6%)	0.431	0.512
	Operating room	154	114 (74.0%)	40 (26.0%)		
Foreign material placed	No	131	124 (94.7%)	7 (5.3%)	89.036	0.000
	Yes	57	17 (29.8%)	40 (70.2%)		
Duration of aortic cross-clamp	≤45 min	41	31 (75.6%)	10 (24.4%)	0.01	0.919
	>45 min	147	110 (74.8%)	37 (25.2%)		
	≤55 min	132	121 (91.7%)	11 (8.3%)	65.651	0.000
	>55 min	56	20 (35.7%)	36 (64.3%)		
	≤60 min	154	138 (89.6%)	16 (10.4%)	96.944	0.000
	>60 min	34	3 (8.8%)	31 (91.2%)		

*CICU* cardiac intensive care unit, *P* < 0.05, Chi squared test

**Table 3:** Comparison of postoperative characteristics of patients with and without surgical site infections

		All (*n* = 188)	Surgical site infection		χ^2^	*P* value
			No (*n* = 141)	Yes (*n* = 47)		
DSC	once	145	135 (93.1%)	10 (6.9%)	110.810	0.000
	More than once	43	6 (14.0%)	37 (86.0%)		
Duration of DSC	≤24 h	89	86 (96.6%)	3 (3.4%)	93.763	0.000
	24 h < DSC ≤48 h	34	31 (91.2%)	3 (8.8%)		
	48 h < DSC ≤72 h	25	16 (64.0%)	9 (36.0%)		
	72 h < DSC ≤96 h	20	5 (25%)	15 (75%)		
	96 h < DSC ≤120 h	11	2 (18.2%)	9 (81.8%)		
	>120 h	9	1 (11.1%)	8 (88.9%)		
ECMO	no	153	137 (89.5%)	16 (10.5)	92.695	0.000
	yes	35	4 (11.4%)	31 (88.6%)		
Duration of mechanical ventilation (h)	≤72 h	82	77 (93.9%)	5 (6.1%)	60.437	0.000
	72 h < time ≤ 120 h	62	50 (80.6%)	12 (19.4%)		
	>120 h	44	14 (31.8)	30 (68.2%)		

*ECMO* extracorporeal membrane oxygenation, *DSC* delayed sternal closure, *P* < 0.05, Chi squared test

**Table 4:** Comparison of length of stay in CICU and hospital of patients with and without surgical site infections

	Surgical site infection		F	t	*P* value
	No (*n* = 141)	Yes (*n* = 47)			
CICU length of stay (d)	7.43 ± 1.451	11.64 ± 2.574	21.970	−10.651	0.000
Hospital length of stay (d)	16.87 ± 1.605	22.26 ± 4.341	97.067	−8.325	0.000

*CICU* cardiac intensive care unit, *P* < 0.05, two independent samples *t*-test

**Table 5:** Multivariate model of risk factors for surgical site infections

Variable	OR	95% CI	*P* value*	Overall forecast percentage
Weight	0.998	0.997–0.999	0.001	90.4%
Age at operation	0.581	0.474–0.711	0.000	

*OR* odds ratio, *CI* confidence interval

*From multivariate logistic regression model

## Discussion

The significant of delayed sternal closure after operating on pediatric cardiac is well reported [14]. For patients with DSC, the decision is at the discretion of the attending surgeon and is typically based on the hemodynamic stability and coagulation status of the patient. Frankly speaking, this is the first study to examine the potential risk factors during the pre-, intra-, and postoperative periods for these SSIs of neonates with DSC. Infection rates after cardiac surgery difference based on the type and complexity of operation [13], but the impact of an DSC on development of SSI is uncertain. Our retrospective study Indicates a significantly higher prevalence of SSI in those underwent with delayed sternal closure. DSC is a risk factor for surgical site infection [7]. It is a complication of median sternotomy [9]. Our rates of SSI (9.7%) and organ/deep SSI (7%) were both higher than the previously published rates by Tabbutt and colleagues (6.7 and 3.9%, respectively) [22]. A reasonable explanation for these differences is that we studied newborns. They did not conduct a systematic analysis of newborns with delayed chest closure.

Surgical incision infection is the result of the interaction of multiple systems. In congenital heart disease surgery, foreign material implantation may cause abnormal immune system challenges, perhaps resulted in SSI. In our study, The possibility of SSI was about 70.2% when the related materials were implanted into the cardiovascular system. Implants may cause changes in hemodynamics, such as turbulence, eddies, etc., thrombosis or bacterial colonization and bacteremia. (Table 2). However, the causal relationship between SSI and implants needs further discussion. In fact [23], according to the literature, children with intracardiac implants are at high risk of infective endocarditis. Whether the trigger factor for infective endocarditis is always a transient bacteraemia is largely accepted. As a general rule, bacteraemia is clinically benign and self-correcting in children who are not suffering from immune system deficiency as well as turbulent cardiac blood flow. As universally known, this system is a very complex network. After congenital heart disease surgery, some children, especially those who experience delayed chest closure, have very poor physiological function, their immune system is on the verge of collapse, and their immune function is relatively weak, so they are prone to infection. The more serious the heart malformation is, the longer the cardiopulmonary bypass time and aortic occlusion time may be.

Our results suggest that SSI can be avoided to a great extent when aortic occlusion time is less than 45 min. When the aortic occlusion time was more than 60 min, the probability of SSI was as high as 91.2%. Therefore, shortening the aortic occlusion time during the operation can significantly reduce the occurrence of SSI. (Table 2). After cardiopulmonary bypass, the body will experience ischemia–reperfusion injury, which has an impact on wound healing. Duration of aortic cross-clamp may affect wound healing.

More and more attention has been paid to the application of ECMO in the treatment of congenital heart disease. At the same time, the associated risks need attention. In the analysis of whether to use ECMO, we found that the prevalence of SSI was about 88.6% when using ECMO. (Table 3). This may be because in the ECMO process, various pipelines are implants, and there may be delayed chest closure, multiple incisions, etc., to promote the occurrence of SSI. Moreover, patients who need ECMO assistance are at the edge of collapse of immune function and prone to SSI.

The healing of incision is the result of the interaction of various systems. The liver provides a variety of proteins, and the immune system is a collective warrior. The older the children are, the more mature their organ functions are and the more stable their functions are. In our study, we found that the probability of SSI was 84.6% when the operative age was less than 1 day. However, when the operative age was between 7 days and 14 days, The possibility of SSI was significantly reduced. In this group, patients more than 14 days old had no SSI (Table 1). The probability of SSI-free status in mature neonates was about 93.2% (Table 1). This may be related to the characteristics of the growth and development of the newborn.

It is particularly important to find a simple way to predict the prevalence of SSI before surgery. In research on preoperative factors, we found that the prevalence of SSI was 87.5% when the body weight was less than 1500 g. However, when the body weight was more than 3000 g, the probability was 96.7% that SSI would not develop (Table 1). This shows that the weight of newborns has a great influence on SSI in DSC patients. The weight was highly correlated with prematurity, and thus excluded from the multivariate model to avoid multicollinearity. Multivariate logistic regression analysis showed that weight and age at surgery were significantly associated with increased prevalence of SSI (OR: 0.998 and 0.581, respectively; 95% CI: 0.997–0.999 and 0.474–0.711, respectively) (Table 5). In this way, we can predict the prevalence of SSI in DSC neonates before surgery.

Infections not only contribute to morbidity and mortality after heart surgery, but also may increase the burden of health care costs of prolonged mechanical ventilation and hospital stay in the DSC group due to the requirement for mechanical ventilation while the sternum remains open. As in our experiment, the hospitalization time and intensive care unit time in patients with SSI were significantly higher than those in the control group (Table 4). In this retrospective study, we found that the prevalence of SSI-free status was 93.9% when the duration of mechanical ventilation was less than 72 h. However, when the duration of mechanical ventilation was more than 120 h, the prevalence of SSI was 68.2% (Table 3). Although the ratio is not high, the probability of SSI increases exponentially as the duration increases. Mechanical ventilation was over after chest closure, thus adding additional days on the ventilator and in the CICU for patients with DSC. It is a factor contributing to prolonged CICU stay. This group also had an increased mortality. Of the three in-hospital deaths in the DSC group, nearly 4.3% (2 of 47) of newborns who developed SSI died. Our study confirms previous observations that even in the current era, SSI remains an important contributor to postoperative complications among neonates with CHD who undergo DSC.

We were particularly interested in the possible association between duration of DSC and prevalence of SSI. Multiple authors have reached variable conclusions regarding this question. A recent retrospective review of adult patients demenstrated that although prolonged delay before sternal closure was related to poor outcome, there was no discrepancy in the frequency of SSI compared with patients with primary closure. Previous pediatric studies have not shown an association between duration of DSC and SSI. There is no study of neonates about those at the moment. However, our study found that the prevalence of SSI absence was 96.6% when the duration of DSC was less than 24 h. However, when the duration of DSC was more than 120 h, the prevalence of SSI was 88.9% (Table 3). In any case, the prevalence of SSI is positively correlated with duration of DSC, and in the study of postoperative factors, when comparing DSC with multiple DSCs, we found that patients with multiple DSC were about 86% more likely to develop SSI (Table 3). This indicates that shortening the duration of DSC and reducing the number of DSCs are important ways to prevent the occurrence of SSI.

These findings may have important clinical implications, because current practice is generally aimed at reducing the duration of DSC.

In our study, the major pathogens identified were coagulase negative Staphylococcus species. Bacteria were the most common pathogenic microorganism. Some pathogens, coagulase-negative staphylococci and Pseudomonas sp., are commonly associated with device-related or other nosocomial infections. Bacterial resistance was usual. Most infections were also given rise to organisms with intrinsic resistance to commonly used antimicrobials. Long and colleagues [24] considered DSC as an independent risk factor for the development of mediastinitis. Guide on antibiotic use for infection prevention in patients with delayed sternal closure is extremely limited. The Society of Thoracic Surgeons (STS) guidelines for rational use of antibiotics in cardiac surgery provide recommendations on both duration and choice of antibiotic, but lack definite recommendations for antibiotic application for patients with DSC [25]. For newborns with DSC, no mention is made. The choice of prophylactic antibiotics varies among surgeons and centers, and the optimal regimen is uncertain. On the other hand, there is a concern for raised prevalence of Gram-negative infections due to usually used prophylactic antibiotic strategies [26, 27]. Previous studies suggested that broad-spectrum antibiotics and/or extended duration of prevention may be used in an attempt to cut down danger of surgical site infections. However, due to the retrospective study, it was difficult to definate whether broad-spectrum antibiotics were used and duration was extended for treatment of known or suspected infection, or whether antibiotics were directed as prevention. The selection of the optimal agent and duration of therapy are vital for the rational use of antibiotics. Based on our study, We strive to reduce the use of broad-spectrum antibiotics and shorten the time of preventive use of antibiotics. Additional multi-institutional research is necessary to form guidelines associated with SSI in neonatal CHD with DSC. In general infections not only lead to prevalence and mortality after heart surgery but also may raise the economic burden of medical expenses of prolonged mechanical ventilation and hospital stay among the DSC group caused by the requirement for mechanical ventilation while the sternum remains open. These findings may have important clinical significance because this is the theoretical basis for shortening the duration of DSC. Through the above analysis, we should try our best to treat this kind of patients within the scope of safety in every link, so as to reduce the pain of children and reduce the economic burden of children’s families. When a patient inevitably has these high-risk factors, we can take some necessary preventive or intervention measures. Such as upgrading the use of antibiotics, the use of combined antibiotics, etc.

### Limitations

This retrospective study is limited by data variables from a single institution. The retrospective research methods further limited observation of factors possibly related to prevalence of SSI, such as the timing of sternal closure. Patients with late infections may not have been observed and excluded. In fact, some data about other risk factors such as perioperative glucose levels, duration of parenteral nutrition, and so on were not recorded and not analyzed in our study. Future study design could make a forward-looking to randomize patients with DSC after cardiothoracic surgery to evaluate the impact of different antibiotic regimens, forming guidelines for rational use of antibiotics.

## Conclusion

Through the above analysis, we draw the following conclusions.

a) Placement of foreign bodies, including a drainage tube, increases the prevalence of SSI in neonatal patients with CHD who undergo DSC. b) The duration of aortic occlusion is less than 45 min, and the effect on SSI is negligible. However, with the prolongation of aortic clamping duration, the probability of developing SSI increased among neonatal CHD patients who underwent DSC. c) The study showed that the use of ECMO could also increase the prevalence of SSI among these patients. d) The age at surgery was closely related to the occurrence of SSI among these neonates as well. The probability of developing SSI was negatively correlated with the age at surgery. e) Compared with full-term infants, the prevalence of SSI is greater in premature infants. f) Weight has a great impact on the probability of SSI. Body weight was negatively correlated with the occurrence of SSI. g) Multivariate logistic regression analysis showed that weight and age at the time of surgery were significantly associated with increased prevalence of SSI. In this way, we can predict the prevalence of SSI in DSC neonates before surgery. h) In any case, the frequency of SSI is positively correlated with duration of DSC.

## Data Availability

The datasets used and/or analysed during the current study are availablefrom the corresponding author on reasonable request. All data generated or analysed during this study are included in this published article.

## Abbreviations

CHD: Congenital heart disease
DSC: Delayed sternal closure
SSIs: Surgical site infections
CICU: Cardiac intensive care unit
STROCSS: Strengthening the Reporting of Cohort Studies in Surgery
CDC: Centers for Disease Control and Prevention
ECMO: Extracorporeal membrane oxygenation
OR: Odds ratio
CIs: Confidence intervals
NHSN: National Healthcare Safety Network
STS: Society of Thoracic Surgeons
MSSA: Methicillin-susceptible *Staphylococcus aureus*
